# Serotonin/GABA receptors modulate odor input to olfactory receptor neuron in locusts

**DOI:** 10.3389/fncel.2023.1156144

**Published:** 2023-04-28

**Authors:** Mingyue Lv, Xiao Xu, Xinyang Zhang, Bo Yuwen, Long Zhang

**Affiliations:** ^1^Department of Agricultural Insects and Pest Control, China Agricultural University, Beijing, China; ^2^Department of Zoology, University of Cambridge, Cambridge, United Kingdom; ^3^Plant Protection Institute, Shandong Provincial Engineering Technology Research Center on Biocontrol for Pests, Jinan, China

**Keywords:** modulation, 5-HT receptor, GABA receptor, olfaction, olfactory receptor neurons, peripheral nervous system

## Abstract

**Introduction:**

Serotonin (5-hydroxytryptamine; 5-HT) and GABA (γ-aminobutyric acid) are involved in the regulation of behaviors in the central nervous system. However, it remains unclear whether they modulate olfaction in the peripheral nervous system, and how they modulate olfaction.

**Methods and results:**

One 5-HT receptor sequence (*Lmig*5-HT2) and one GABA receptor sequence (*Lmig*GABAb) were identified in locust antennae by transcriptome analysis and polymerase chain reaction experiments. *In situ* hybridization localized *Lmig*5-HT2 to accessory cells, while *Lmig*GABAb was localized to olfactory receptor neurons (ORNs) in locust chemosensilla. Single-unit electrophysiological recordings combined with RNA interference (RNAi) experiments indicated ORNs of locusts with knockdown of *Lmig*5-HT2 (ds-*Lmig*5-HT2) and *Lmig*GABAb (ds-*Lmig*GABAb) to some odors had significantly higher responses than wild-type and control locusts in the dose-dependent responses. Moreover, the gaps between the responses of ORNs of RNAi ones and those of wild-type and ds-GFP enlarged with an increase in concentrations of odors.

**Discussion:**

Taken together, our findings suggest that 5-HT, GABA, and their receptors exist in the insect peripheral nervous system and that they may function as negative feedback to ORNs and contribute to a fine-tuning mechanism for olfaction in the peripheral nervous system.

## 1. Introduction

Olfactory information processing involves a cascade of neural events that initiate at the level of peripheral olfactory organs and propagate through ascending pathways to the central nervous system (CNS). The information processed in olfactory streams is regulated by descending control systems, such as neuromodulatory systems; this process has been studied in the CNS in both vertebrates (McLean et al., 1993; Dugué and Mainen, 2009; Fletcher and Chen, 2010; Liu et al., 2012; Kapoor et al., 2016) and invertebrates, including insects (Gatellier et al., 2004; Dacks et al., 2008, 2009; Kloppenburg and Mercer, 2008). However, the role of neuromodulation in the processing of odor responses at the peripheral level remains poorly understood.

Antennae, the peripheral olfactory organs of insects, mainly function to acquire odor cues. In the peripheral olfactory process, odor molecules enter the lumen of hair-like organs called chemosensilla on insect antennae, where the molecules are bound and transported by odorant-binding proteins (OBPs). The odorants or odorant/OBP complexes, then, arrive at the dendrites of olfactory receptor neurons (ORNs), are bound at the membrane by odorant receptors (ORs) in chemosensilla, and evoke a membrane potential change, sending signals to the CNS (Hildebrand and Shepherd, 1997; Clyne et al., 1999; Dobritsa et al., 2003; Hallem and Carlson, 2006; Wang and Anderson, 2010; Leal, 2013).

Odorant receptors (ORs) either form ion channels directly with a co-receptor or couple with other proteins to perform metabotropic signal transduction (Sato et al., 2008; Wicher et al., 2008). In addition, OBPs are secreted by accessory cells surrounding ORNs and are commonly suggested to solubilize and transport non-soluble exogenous organic compounds to ORs (Pelosi et al., 2006; Laughlin et al., 2008). Investigating neuromodulation in antennae is crucial for understanding its role in the peripheral nervous system.

Serotonin (also known as 5-hydroxytryptamine; 5-HT) is a ubiquitous neuromodulator that is found throughout phylogeny and alters olfactory function in the CNS (Dierick and Greenspan, 2007; Ganesh et al., 2010; Johnson et al., 2011; Albin et al., 2015). It can directly suppress projection neuron responses to odors (Kloppenburg and Hildebrand, 1995; Petzold et al., 2009; Zhang and Gaudry, 2016; Gaudry, 2018). Moreover, 5-HT enhances the response of inhibitory local interneurons, GABAergic neurons, which indirectly results in reduced neurotransmitter release from ORN terminals via GABA_b_ receptor-dependent presynaptic inhibition (Dacks et al., 2009; Dugué and Mainen, 2009; Petzold et al., 2009). Nevertheless, very few studies have focused on the role of 5-HT at the peripheral level (Dolzer et al., 2001). 5-HT is found in the hemolymph of insects (Lange et al., 1989; Siju et al., 2008; Zhukovskaya and Polyanovsky, 2017). In addition, 5-HT-immunoreactive fibers and putative 5-HT receptor genes have been identified in the antennae of mosquitoes (Siju et al., 2008; Pitts et al., 2011). These findings suggest the possibility that serotonergic modulation occurs in antennae.

To determine if there is a feedback mechanism in the olfactory peripheral nervous system of insects, immunohistochemistry was used to determine the localization of 5-HT and GABA in locust antennae. We, then, identified 5-HT and GABA receptors in locust antennae and characterized their expression patterns with dual-color fluorescence *in situ* hybridization experiments. We used a combination of single-unit electrophysiology and RNA interference (RNAi) techniques to reveal the functions of 5-HT and GABA receptors in ORNs. Our results suggest that, in the peripheral nervous system, 5-HT/GABA and their receptors may independently function to some extent as negative feedback in the response of ORNs to high concentrations of odors.

## 2. Materials and methods

### 2.1. Animals

*Locusta migratoria* (Orthoptera) was raised under crowded conditions in the Department of Entomology, China Agricultural University, with a relative humidity of 60% and a temperature of 28–30°C, under a photoperiod of 18:6 h (light:dark). Locusts were fed fresh wheat seedlings daily. Intact antennae were dissected using forceps and stored at −80°C until mRNA extraction, immunohistochemistry, and *in situ* hybridization.

### 2.2. Identification of 5-HT-/GABA-related genes in antennae

We used a collection of insect 5-HT-/GABA-related genes (Supplementary Tables S1–S3) to perform tblastx queries with a cutoff of 10^−5^ against our database using the *L. migratoria* antenna transcriptome. Identified hits, indicating candidate-related genes, were used to re-tblast against the National Center for Biotechnology Information database to verify gene identities. The phylogenetic analysis of 5-HT/GABA receptors was performed with multiple aligned sequences using the maximum likelihood distance method, with a bootstrap value of 1,000, in MEGA6 for Windows.

### 2.3. *In situ* hybridization

Templates of *Lmig*OBP1, *Lmig*ORco, *Lmig*5-HT_2_, *Lmig*GABA_b_, *Lmig*TPH, and *Lmig*AADC were generated by standard polymerase chain reaction (PCR) using gene-specific primer pairs (Supplementary Table S4). Digoxigenin (DIG)- or biotin (BIO)-labeled antisense probes were generated from linearized recombinant pGem-T Easy plasmids using the T7/SP6 RNA transcription system (Roche, Basel, Switzerland), following the recommended protocols. RNA *in situ* hybridization was performed according to previously reported procedures (Xu et al., 2017). In brief, antennae were dissected and embedded in a freezing medium (Tissue-Tek O.C.T. Compound; Sakura Finetek Europe). Sections (12 μm) were prepared in the same way as for immunohistochemistry. After a series of fixing and washing procedures, 100 μL of hybridization solution (Boster) containing RNA probes was applied to the tissue sections. After adding coverslips, slides were incubated in a humid box at 55°C overnight. After hybridization, slides were washed two times for 30 min in 0.1× saline-sodium citrate at 60°C, treated with 1% blocking reagent (Roche) in PBST for 30 min at room temperature, and then incubated for 30 min with an anti-DIG alkaline phosphatase-conjugated antibody (Roche). DIG-labeled probes were visualized using the anti-DIG alkaline phosphatase-conjugated antibody in combination with HNPP/Fast Red (Roche). For BIO-labeled probes, the TSA Kit (PerkinElmer, Waltham, MA, United States), including a streptavidin–horseradish peroxidase conjugate and fluorescein tyramide as a substrate, was used. Images were captured on an Olympus BX45 confocal microscope and analyzed using FV1000 software (Olympus). Images were not altered except for the uniform adjustment of brightness or contrast within a single figure.

### 2.4. RNAi

Double-stranded RNA (ds-RNA) was synthesized following the manufacturer's instructions. In brief, PCR products (for primer pairs see Supplementary Table S4) were amplified with T7 promoter conjugated primer and then purified using the Wizard^®^ SV Gel and PCR Clean-Up System (Promega, Madison, WI, United States) as templates for *in vitro* transcription. ds-RNA was synthesized with T7 RiboMAX^TM^ Express RNAi System (Promega), diluted to 1,000 ng/μL with ddH_2_O, and stored at −20°C. Target ds-RNA (10 μg) was delivered into each locust dorsal vessel through the inter-segmental membrane of nymphs on the first day of the fifth instar, using an IM-9B microinjector (Narishige, Tokyo, Japan) equipped with a glass capillary. ds-green fluorescent protein (GFP) was microinjected as a control group. The treated locusts were raised normally like wild-type insects. RNA silencing efficiency was checked on post-injection day 3. The number of biological replicates was at least 4, and the sample number in each replicate was at least 2. All RNAi-treated insects that were used for single sensillum recordings (SSRs) were also checked after recording to confirm the silencing efficiency.

### 2.5. Semi-quantitative real-time (RT)-PCR

Semi-quantitative RT-PCR was used to check the expression of target genes and investigate the silencing efficiency of gene-specific RNAi. The biological replicate was 3, and there were at least two samples in each replicate. In brief, 1 μg total RNA from various RNAi treatments and control tissues were transcribed into cDNA. For each independent PCR, gene-specific primer pairs were designed (see Supplementary Table S4), and the equivalent amount of cDNA was used as a template for amplification (TaKaRa Ex Taq, TaKaRa, Shiga, Japan). The following PCR cycling parameters were used: initial denaturation at 94°C for 5 min; 35 cycles of 94°C, 30 s; 50–60°C, 30 s (different genes had different annealing temperatures, see Supplementary Table S3); and 72°C, 1 min; followed by a final extension at 72°C for 10 min.

### 2.6. qPCR

The template of real-time quantitative PCR (QPCR) was a transcript from the same RNA samples of transcriptome sequencing using M-MLV reverse transcriptase (Promega, United States), according to the manufacturer's instructions. We designed the QPCR primers with 100–250 bp product primers from the unigene sequences. The primers were checked with normal PCR (TaKaRa, Dalian, Liaoning, China) and sequencing to verify product correction and no primer dimers. The 2^−ΔΔCT^ method was used to qualify the relative expression levels of each gene. The expression level of genes was normalized by reference genes *Rp49*. The 20 μl reaction volume (including 10 μl SuperReal PreMix, Tiangen, Beijing, China) for qPCR was performed on an ABI QuantStudio 6 Flex (United States), with the following PCR program: 15 min at 95 °C, 40 cycles of 10 s at 95 °C, 30 s at 60 °C, and at last 60 °C to 95 °C to perform melting curve analysis and evaluate the specificity of the real-time PCR products. Each sample was performed with three technical replicates.

### 2.7. Single-unit electrophysiological experiments

Our *in situ* hybridization experiments showed that ORNs and accessory cells of sensilla on the distal sixth segment of antennae expressed *Lmig*ORco, *Lmig*OBP1, *Lmig*5-HT_2_, and *Lmig*GABA_b_. Moreover, scanning electron microscopy indicated that this sensillum was a basiconic sensillum; we, therefore, named it Ba6. Ba6 was used for the following SSR experiments. SSRs and chemical stimulations were performed on the antennae of fifth instar nymphs. Each locust or isolated antenna was mounted with tape and plasticine on a glass slide, and one of its antennae was immobilized with tape and plasticine on a piece of coverslip. Tungsten electrodes were sharpened electronically with 10% NaNO_2_ under a microscope. The recording electrode was inserted into the base of the basiconic sensillum using a motorized micromanipulator (CFT-8301D, C.M.D.T., China), and a ground electrode was inserted into the head. The stimulation duration was 1 s. The recording electrode was connected to a 10× universal AC/DC amplifier (Syntech, Venlo, the Netherlands). The recording signals were collected on an Intelligent Data Acquisition Controller (IDAC-4, Syntech) and were viewed on a personal computer. AUTOSPIKE (Syntech) software was used to quantify the neuronal responses recorded for 1 s before and after each stimulation, and the duration between the maximum point of frequency after stimulation to a 50% reduction of the response.

### 2.8. Chemicals and preparation

We chose benzaldehyde, phenylacetonitrile, and guaiacol to investigate during the SSR experiments. All three of these chemicals have been reported in the body odors of gregarious nymphs in migratory locusts, and they have been suggested to have important roles in the density-dependent phase change in locusts (Wei et al., 2017). Chemicals with the highest purity were used for SSRs; these are presented in Supplementary Table S5. The working solutions were prepared using mineral oil as the solvent.

### 2.9. Statistical analyses

Data from extracellular recordings were analyzed using the one-way analysis of variance (ANOVA) and Tukey's honest significant difference (HSD) *post-hoc* test in GraphPad Prism 7 (GraphPad Software, San Diego, CA, United States).

## 3. Results

### 3.1. 5-HT and its receptors exist under the olfactory sensilla of antenna

**Figure 1:**
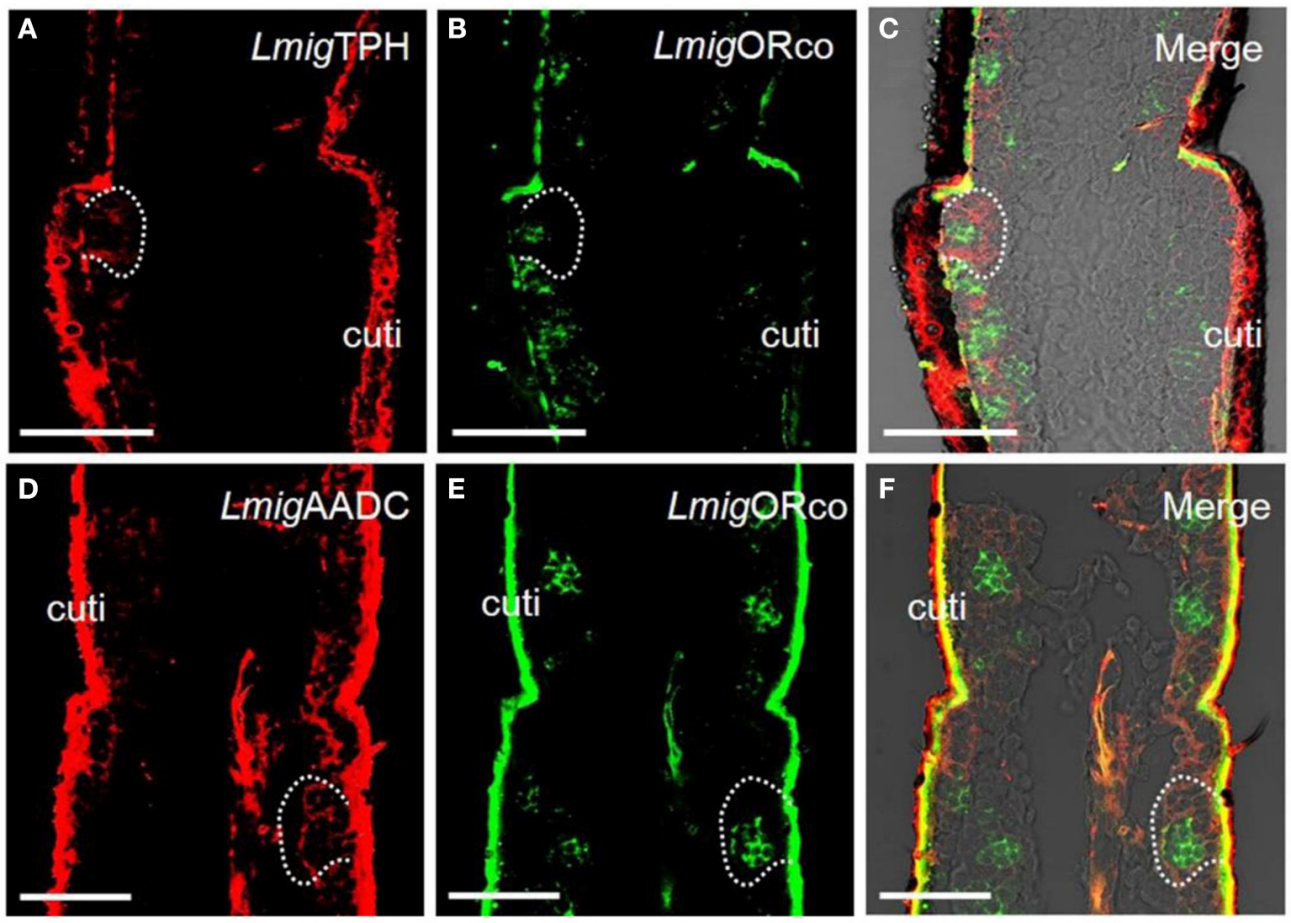
Localization of 5-HT using antiserum against 5-HT and localization of two key enzymes for 5-HT biosynthesis by dual-color fluorescence *in situ* hybridization in locust antennae. **(A)** The enzyme *Lmig*TPH was stained by an antisense probe against *Lmig*TPH RNA with digoxigenin (DIG), red. **(B)** Odorant receptor co-expressed protein (*Lmig*ORco) was stained by an antisense probe against *Lmig*ORco RNA with biotin (BIO), green. **(C) A** and **B** merged with the brightfield image. **(D)** The enzyme *Lmig*AADC was stained by an antisense probe against *Lmig*AADC RNA with DIG, red. **(E)** *Lmig*ORco was stained by an antisense probe against *Lmig*ORco RNA with BIO, green. **(F) D** and **E** merged with the brightfield image. Ba, basiconic sensilla; Tr, trichoid sensilla; Co, coeloconic sensilla; cuti, cuticle; tra, trachea. Scale bar: 100 μm.

The genes encoding the putative key enzymes involved in the 5-HT synthesis, tryptophan hydroxylase (*LmigTPH*), and aromatic L-amino acid decarboxylase (*LmigAADC*, MN531682) were searched from the transcriptome database of locust antennae using BLAST. A phylogenetic tree was constructed (Supplementary Figure S1A), and PCR results revealed that the transcripts of these two genes existed in locust antennae (Supplementary Figure S1B). This finding indicates that 5-HT might be able to be biosynthesized in antennae. Furthermore, we synthesized *LmigTPH* or *LmigAADC* RNA antisense probes with DIG (red) to conduct dual-color fluorescence *in situ* hybridization experiments with *LmigORco* using a BIO-labeled probe (green). The OR co-expression receptor (ORco) is expressed in every ORN; thus, cells labeled by the *LmigORco* antisense probe can be taken to be ORNs. The antisense probes against *LmigTPH* or *LmigAADC* (red) and antisense probes against *LmigORco* (green) did not overlap fully, but they occurred in close proximity to sparse cell clusters (Figures 1A–F). Taken together, our results indicate that 5-HT exists around, but not in, ORNs in locust antennae.

Because 5-HT was observed in the antennae, we hypothesized that there may also be 5-HT receptors in the antennae to bind with the 5-HT. To examine whether there are 5-HT receptors in locust antennae, we first searched for the putative receptor genes in the transcriptome database of locust antennae using BLAST. One partial gene sequence was considered a putative 5-HT receptor gene. The phylogenetic tree indicated that the partial sequence belonged to one insect 5-HT receptor (Supplementary Figure S4A), namely, *Lmig*5-HT_2_ (MN531679). We, then, cloned the putative gene from locust antennae using PCR (Supplementary Figure S4B), thus indicating the existence of these three genes in locust antennae.

### 3.2. Accessory cells, but not ORNs, express 5-HT_2_ receptors in chemosensilla

**Figure 2:**
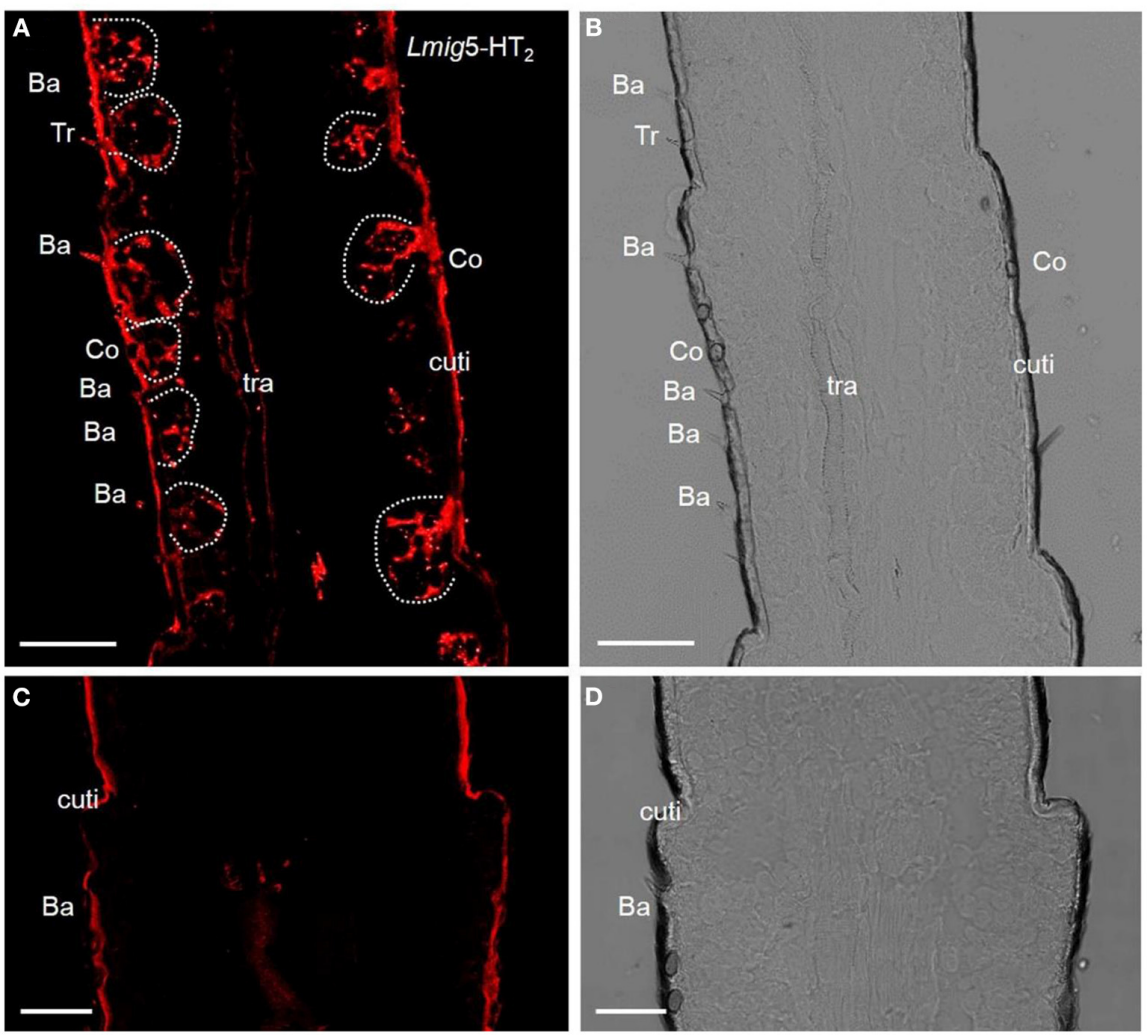
5-HT receptors in locust antennae. **(A)** Single-color fluorescence *in situ* hybridization on the fifth to seventh segments with an antisense probe against *Lmig*5-HT_2_ RNA with DIG, red. **(B)** Blank control. Dotted circles indicate cell clusters. **(C, D)** Negative control without an antisense probe against *Lmig*5-HT_2_ RNA with DIG, red. Ba, basiconica sensilla; Tr, trichoid sensilla; cuti, cuticle. Scale bar: 100 μm.

The preliminary analysis of single-color fluorescent signals of locust 5-HT_2_ receptors (*Lmig*5-HT_2_) demonstrated that a subset of distinct antennal cells was present in each section (Figure 2A). Cell clusters labeled by the antisense probes were observed within all types of olfactory sensilla such as basiconic, trichoid, and coeloconic when checked in the same sections with a plain lens (Figure 2B). In negative controls (Figures 2C, D), autofluorescence in locust cuticle was observed as in many previous studies (Xu et al., 2013; Zhang et al., 2017).

**Figure 3:**
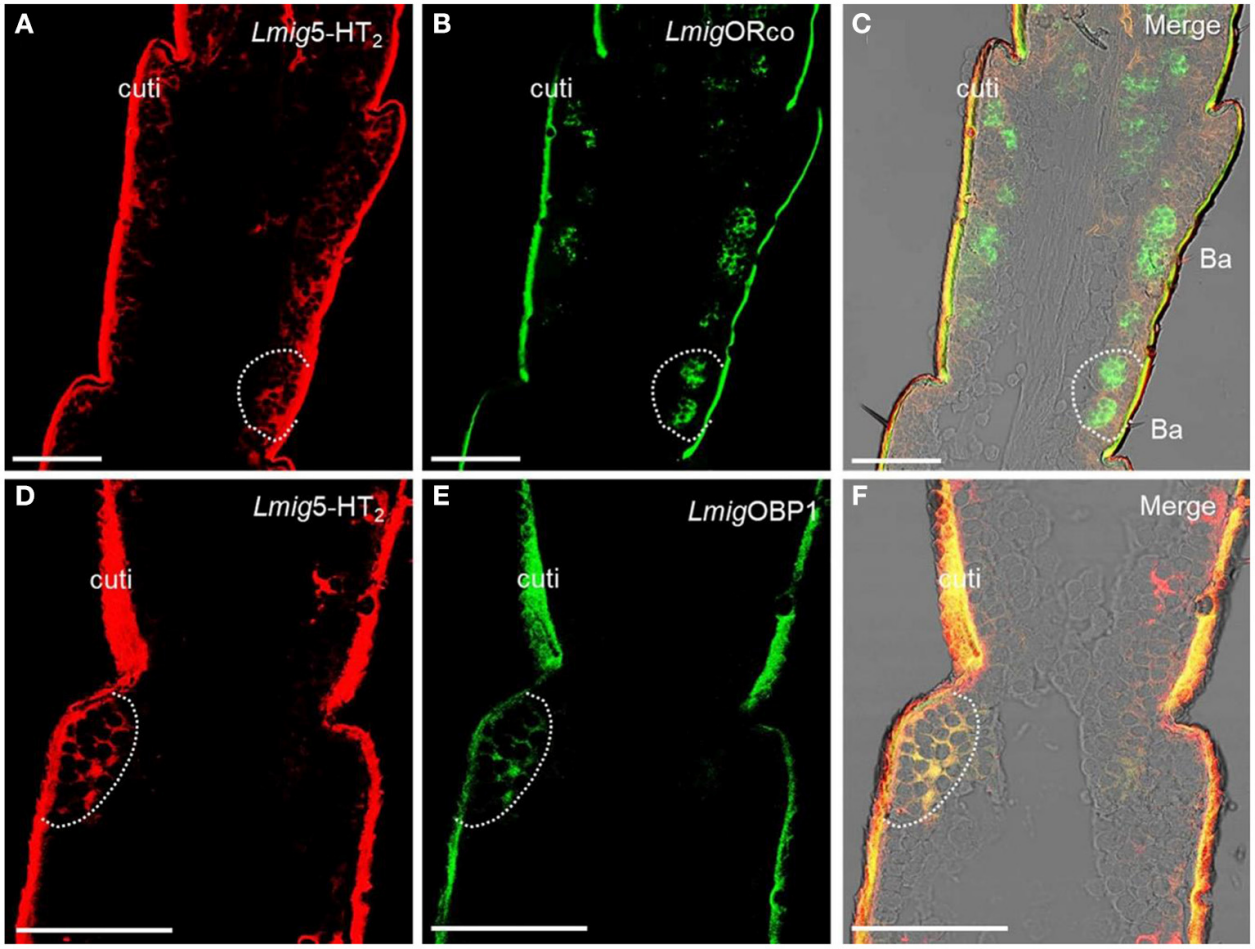
Localization of *Lmig*5-HT_2_ by dual-color fluorescence *in situ* hybridization experiments in locust antennae. **(A)** Antennal cryosection stained by an antisense probe against 5-HT_2_ RNA with DIG, red. **(B)** Antennal cryosection stained by an antisense probe against *Lmig*Orco RNA with BIO, green. **(C) A** and **B** merged with the brightfield image. **(D)** Antennal cryosection stained by an antisense probe against 5-HT_2_ RNA with DIG, red. **(E)** Antennal cryosection stained by an antisense probe against *Lmig*OBP1 RNA with BIO, green. **(F) D** and **E** merged WITH the brightfield image. Cuti, cuticle. Scale bars: 100 μm.

Odorant-binding proteins (OBPs) and ORs in the olfactory sensilla are two protein families which are involved in peripheral odorant detection. OBPs are secreted by accessory cells, and *Lmig*OBP1 is widely expressed in accessory cells of antennal olfactory sensilla (Jin et al., 2005), whereas Orco is expressed in apparently all ORNs expressing ORs but not ionotropic receptors (IRs) (Yang et al., 2011). Here, *Lmig*Orco can be employed as a marker for ORNs and *Lmig*OBP1 as an indicator of accessory cells. Next, we used *Lmig*OBP and *Lmig*Orco antisense probes to detect *Lmig*5-HT_2_ in accessory cells or ORNs. To distinguish the cell type(s) that expressed *Lmig*5-HT_2_, we carried out dual-color fluorescence *in situ* hybridization experiments, using the 5-HT_2_ RNA antisense probe with DIG (red color) and *Lmig*Orco or *Lmig*OBP1 with BIO (green color). The antisense probes against *Lmig*5-HT_2_ (Figure 3A, red color) and *Lmig*Orco (Figure 3B, green color) did not overlap fully but were observed in close proximity to sparse cell clusters (Figure 3C). Conversely, *Lmig*5-HT_2_ (Figure 3D, red color) and *Lmig*OBP1 (Figure 3E, green color) overlapped fully with one another (Figure 3F). In addition, the cell cluster circled by the dashed line was in a basiconic sensillum situated in the sixth segment, known as Ba6 (Supplementary Figure S3A, red square). Together, these observations indicate that *Lmig*5-HT_2_ is not co-expressed with *Lmig*Orco in ORNs but it is co-expressed with *Lmig*OBP1 in accessory cells neighboring ORNs.

### 3.3. GABA and its receptor *Lmig*GABA_*b*_ exist in locust antennae

Serotonergic modulation in the CNS leads to inhibition via GABA_b_ receptors (Dacks et al., 2009; Petzold et al., 2009). In the present study, we wanted to investigate whether GABA receptors exist in the peripheral nervous system. We analyzed the antennal transcriptome and identified a putative GABA receptor gene. The phylogenetic tree (Supplementary Figure S2A, Supplementary Table S2) indicated that this gene belonged to the clade of the GABA_b_ receptor, named *Lmig*GABA_b_ (MN531681). Using PCR, we revealed that this gene existed in locust antennae (Supplementary Figure S2B).

**Figure 4:**
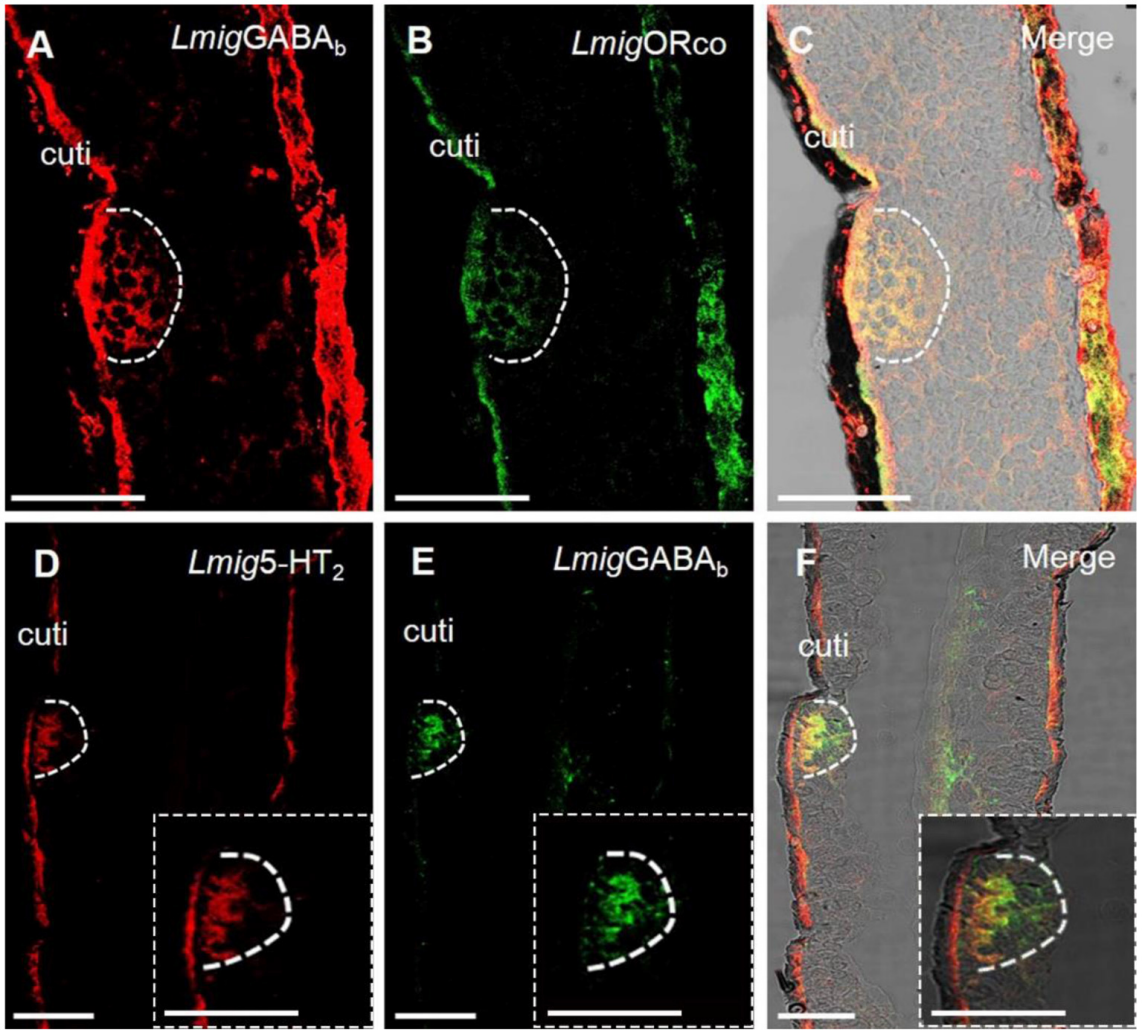
Localization of *Lmig*GABA_b_ by dual-color fluorescence *in situ* hybridization experiments in locust antennae. **(A)** Antennal cryosection stained by an antisense probe against *Lmig*GABA_b_ RNA with DIG, red. **(B)** Antennal cryosection stained by an antisense probe against *Lmig*Orco RNA with BIO, green. **(C) A** and **B** merged with the brightfield image. **(D)** Antennal cryosection stained by an antisense probe against 5-HT_2_ RNA with DIG, red. **(E)** Antennal cryosection stained by an antisense probe against *Lmig*GABA_b_ RNA with BIO, green. **(F) D** and **E** merged with the brightfield image. Cuti, cuticle. Scale bars: 100 μm. The dashed box in the lower right corner of **(D–F)** shows the enlarged staining result.

Dual-color fluorescence *in situ* hybridization experiments demonstrated that the cells labeled by antisense probes against *Lmig*GABA_b_ with DIG (red color) were also labeled by antisense probes against *Lmig*Orco with BIO (green color) (Figures 4A–C). The findings suggest that *Lmig*GABA_b_ is expressed in ORNs. Moreover, dual-color fluorescence *in situ* hybridization to explore the localization relationship between *Lmig*GABA_b_ and *Lmig*5-HT_2_ showed that *Lmig*GABA_b_-expressing cells (green color) were surrounded by cells labeled by *Lmig*5-HT_2_ (red color) in sensilla basiconica in the antennae (Figures 4D–F). The cell cluster circled by the dashed line in Figure 4 is found in a basiconic sensillum named as Ba6, which is located closest to the end of the fifth segment of the antennae. This cell cluster can be marked jointly by *Lmig*5-HT_2_, *Lmig*GABA_b_, and *Lmig*Orco probes pairwise (Figures 3A–C, 4A–F). In addition, dual-color fluorescence *in situ* hybridization experiments had shown that *Lmig*5-HT_2_ and *Lmig*OBP1 overlapped fully in the same sensillium' cell cluster (Figures 3D–F). In summary, these observations indicate that *Lmig*5-HT_2_, *Lmig*GABA_b_, *Lmig*Orco, and *Lmig*OBP1 express in the same sensillum. Among them, *Lmig*5-HT_2_ and *Lmig*OBP1 are expressed in the same cells (accessory cells), and *Lmig*GABA_b_ and *Lmig*Orco are expressed in common ORNs.

**Figure 5:**
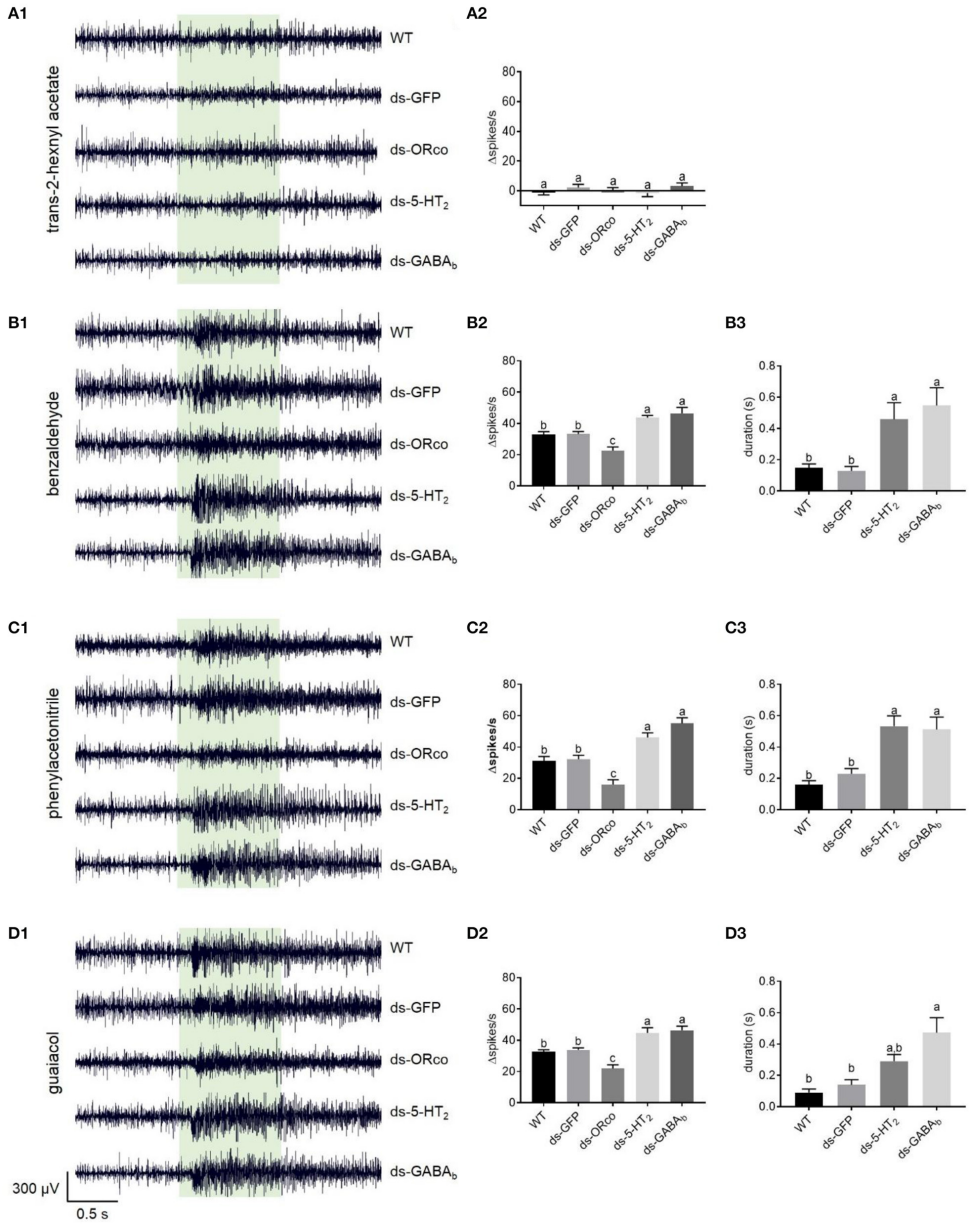
Responses to odorants at 1% (v/v) concentration of ORNs in the antennal basiconic sensilla of wild-type locusts and locusts microinjected with ds-GFP, ds-*Lmig*Orco, ds-*Lmig*5-HT_2_, or ds-*Lmig*GABA_b_. **(A1–D1)** Response traces of Ba6 neurons to trans-2-hexenyl acetate, benzaldehyde, phenylacetonitrile, and guaiacol. **(A2–D2)** Quantification of the mean changes of all spikes (Δspikes/s) in the 1 s before and after the stimulus of the Ba6 neurons to odorants in wild-type locusts and locusts microinjected with ds-GFP, ds-*Lmig*Orco, ds-*Lmig*5-HT_2_, or ds-*Lmig*GABA_b_. **(B3–D3)** Quantification of the duration from the maximum point of frequency after simulation to a 50% reduction of the response to odorants in the treated locusts. The green box indicates the stimulus duration (1 s). WT, wild-type locusts; ds-GFP, locusts injected with ds-GFP; ds-Orco, locusts injected with ds-*Lmig*Orco; ds-5-HT_2_, locusts injected with ds-*Lmig*5-HT_2_; ds-GABA_b_, locusts injected with ds-*Lmig*GABA_b_. Values are the mean ± SEM, *n* = 5–8 per group. For variables with the same letter above the columns, the difference was not statistically significant. For variables with different letters, the difference was statistically significant (*p* < 0.05, one-way ANOVA with Tukey's *post-hoc* tests).

### 3.4. ORNs respond robustly to odorants by knockdown of *Lmig*5-HT_2_ or *Lmig*GABA_*b*_ in antennae

We concluded that *Lmig*5-HT_2_, *Lmig*GABA_b_, *Lmig*Orco, and *Lmig*OBP1 express in the Ba6, which we then chose as the target sensillum to conduct a single-unit electrophysiological experiment (SSR), to elucidate the roles of *Lmig*5-HT_2_ and *Lmig*GABA_b_ in olfaction in antennae. We performed microinjections of ds-RNA of target genes to knock down the expression levels of *Lmig*5-HT_2_ or *Lmig*GABA_b_, thus creating locusts with knockdown of *Lmig*5-HT_2_ and *Lmig*GABA_b_ genes, which we named ds-*Lmig*5-HT_2_ and ds-*Lmig*GABA_b_, respectively. We used locusts that were injected with ds-RNA of GFP as the negative control and locusts injected with *Lmig*Orco as the positive control. Compared with the antennae of wild-type locusts (wt), PCR experiments revealed specific and significant decreases in the levels of *Lmig*5-HT_2_ mRNA in ds-5-HT_2_ locusts, *Lmig*GABA_b_ mRNA in ds**-**GABA_b_ locusts, or *Lmig*Orco mRNA in ds-Orco locusts (Supplementary Figures S3B, S3C). Conversely, there were no changes in the expression levels of these three target genes in the ds-GFP locusts.

The responses of Ba6 neurons to odorants in the ds-*Lmig*5-HT_2_, ds-*Lmig*GABA_b_, ds-*Lmig*Orco, ds-GFP, and wild-type locusts were examined using SSR. The ORNs in Ba6 were not activated by trans-2-hexenyl acetate at a concentration of 1% in wild-type locusts (Supplementary Figure 5A1). Similarly, the targeted neurons in the ds-*Lmig*5-HT_2_, ds-*Lmig*GABA_b_, ds-*Lmig*Orco, and ds-GFP locusts showed no response to 1% trans-2-hexenyl acetate. As expected, the mean changes of all spikes (Δspikes/s) in the 1 s before and after stimulation were not significantly different among the different types of locusts (Supplementary Figure 5A2).

Conversely, benzaldehyde (Supplementary Figure 5B1), phenylacetonitrile (Supplementary Figure 5C1), and guaiacol (Supplementary Figure 5D1) at concentrations of 1% evoked distinct excitement in the Ba6 neurons of wild-type locusts. These three odorants are important body volatiles of gregarious nymphs in migratory locusts. The changed spike number in the Ba6 neurons of wild-type locusts was not significantly different from that of ds-GFP locusts in response to these three odorants at 1% concentration. However, it was significantly lower than those in the Ba6 neurons of ds*-Lmig*5-HT_2_ and ds-*Lmig*GABA_b_ locusts, which were not significantly different from one another. Additionally, the Δspikes/s of Ba6 neurons in ds*-Lmig*5-HT_2_, ds-*Lmig*GABA_b_, ds-GFP, and wild-type locusts were significantly greater than those in the ds-*Lmig*Orco locusts in response to these three odorants (Supplementary Figures 5B2–D2). Moreover, we defined the duration from the maximum point of frequency after stimulation to a 50% reduction of the response as the response duration of Ba6 neurons. The response durations of Ba6 neurons in wild-type and ds-GFP locusts were significantly shorter than those in ds*-Lmig*5-HT_2_ and ds-*Lmig*GABA_b_ locusts in response to benzaldehyde and phenylacetonitrile (Supplementary Figures 5B3–D3), although guaiacol evoked a significantly longer response of Ba6 neurons between the ds-GFP and ds-*Lmig*GABA_b_ locusts but not the ds-*Lmig*5-HT_2_ locusts. These findings suggest that the response duration of ORNs may be prolonged when *Lmig*GABA_b_ is knocked down.

**Figure 6:**
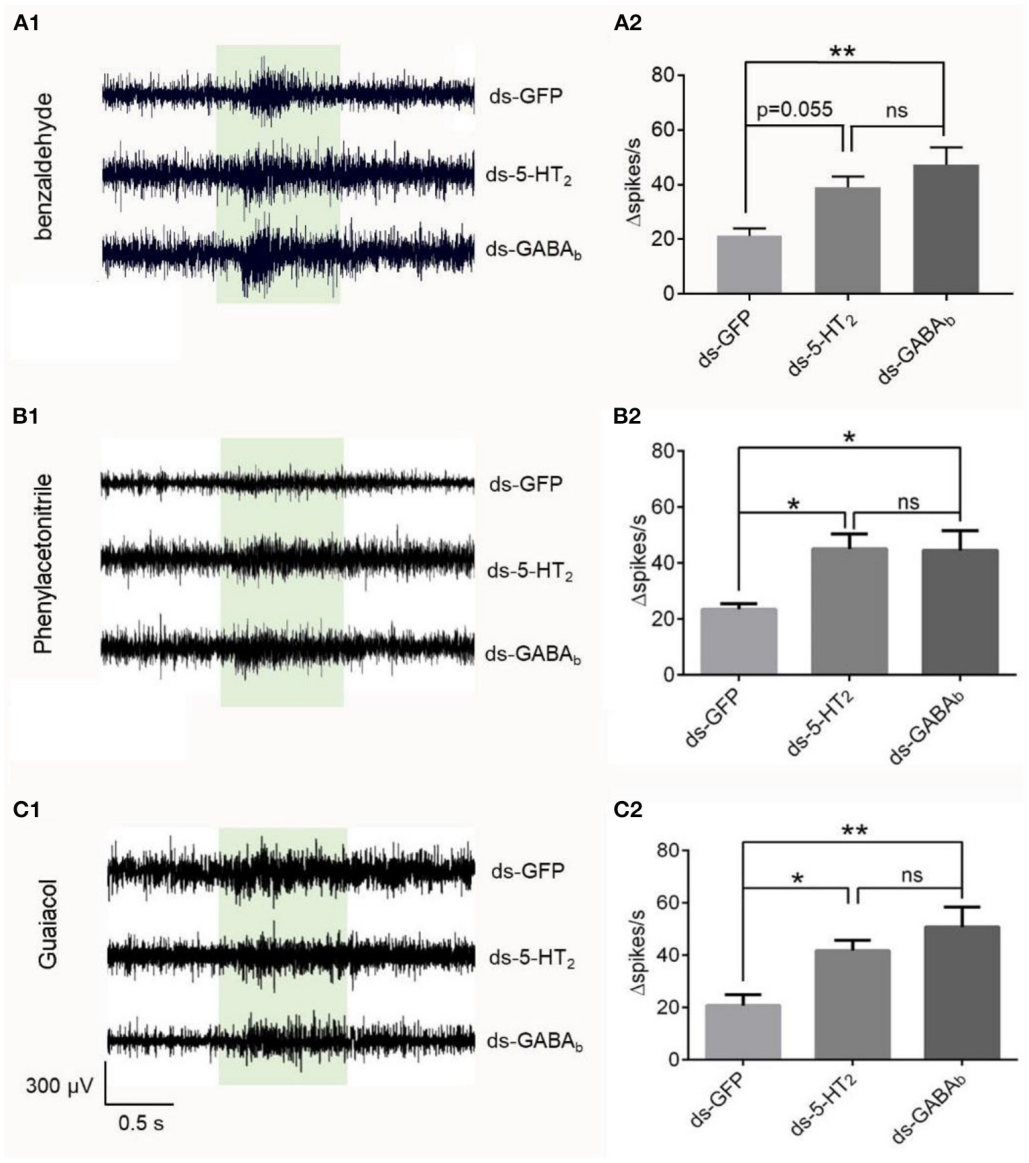
Responses to odorants at 1% (v/v) concentration of ORNs in isolated antennal basiconic sensilla of locusts microinjected with ds-GFP, ds-*Lmig*5-HT_2_, or ds-*Lmig*GABA_b_. **(A1–C1)** Response traces of Ba6 neurons to benzaldehyde, phenylacetonitrile, and guaiacol. **(A2**–**C2)** Quantification of the mean changes of all spikes (Δspikes/s) in the 1 s before and after the stimulus of the Ba6 neurons to odorants in locusts microinjected with ds-GFP, ds-*Lmig*5-HT_2_, or ds-*Lmig*GABA_b_. ^*^p < 0.05, ^**^p < 0.01. ns, no significant difference.

To check if the effect originated inside the antenna, we used isolated antenna to repeat the SSR experiments combined with RNAi. We found that the changed spike number in the Ba6 neurons of ds-GFP locusts was significantly different from that of ds-GABA_b_ locusts in response to benzaldehyde, phenylacetonitrile, and guaiacol at 1% concentration (Figure 6). In addition, there was no significant difference between ds-5-HT_2_ and ds-GABA_b_ locusts in response to the chemical. Although the difference is not significant, there was also a trend that the response evoked by benzaldehyde in the Ba6 neurons of ds-5-HT_2_ locusts is higher than the ds-GFP locusts (*p* = 0.055). The above results suggest that 5-HT_2_ and GABA_b_ receptors at least play partially the role of negative feedback to ORNs of antennae to odors independently.

Therefore, the downregulation of *Lmig*5-HT_2_ or *Lmig*GABA_b_ resulted in a significant amplification of odor-evoked responses, as represented by Δspikes/s and the response duration of ORNs. This suggests that *Lmig*5-HT_2_ and *Lmig*GABA_b_ may provide negative feedback in local ORNs when they respond to odorants.

### 3.5. Downregulation *Lmig*5-HT_2_ or *Lmig*GABA_*b*_ ORNs respond dose-dependently to odorants

**Figure 7:**
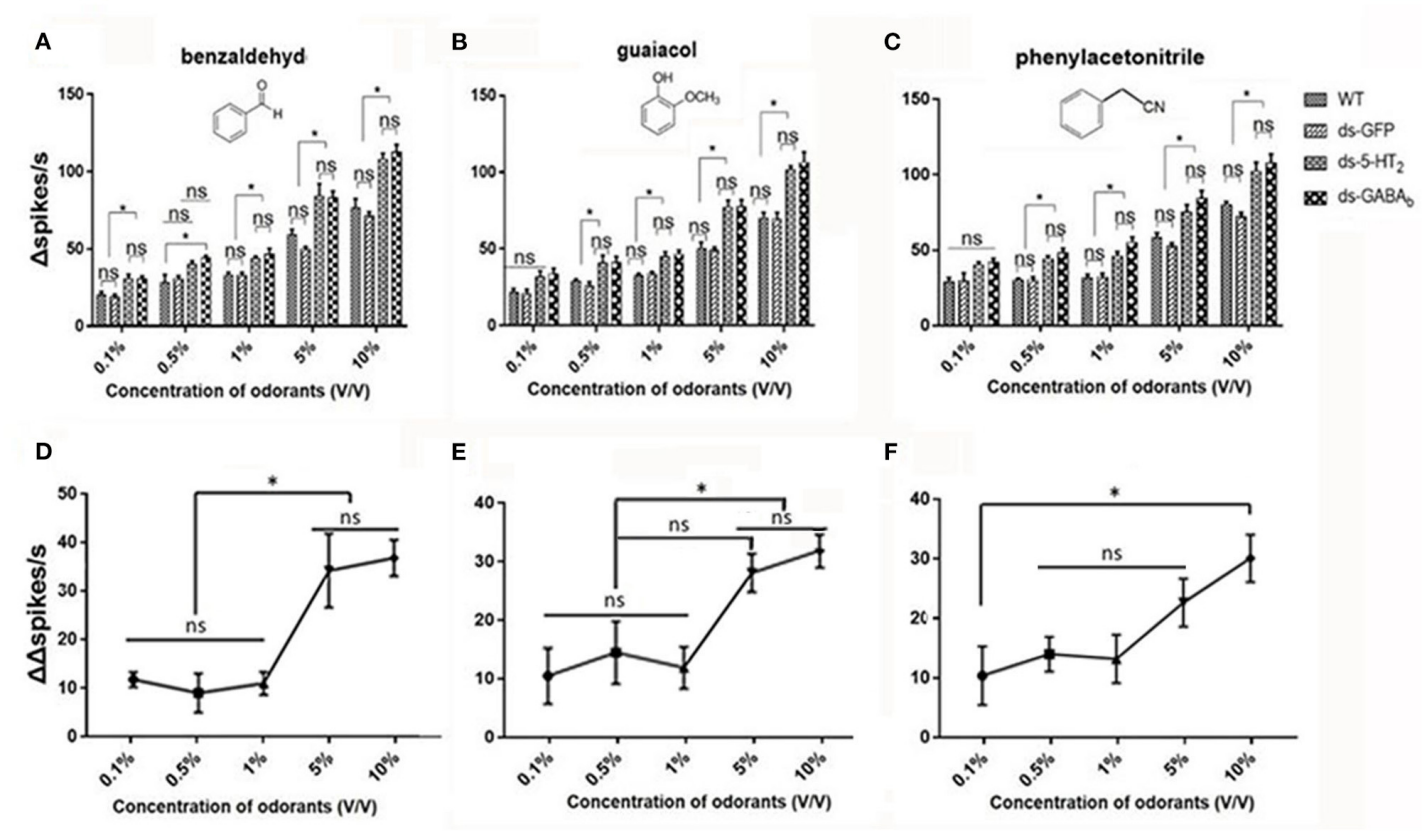
ORNs in the antennal basiconic sensilla of wild-type, ds-GFP, ds-5-HT_2_, and ds*-*GABA_b_ locusts have dose-dependent responses to odorants. **(A)** The Δspikes/s numbers of Ba6 ORNs of locusts (WT, wild-type locusts; ds-GFP, locusts injected with ds-GFP; ds-5-HT_2_, locusts injected with ds-*Lmig*5-HT_2_; ds-GABA_b_, locusts injected with ds-*Lmig*GABA_b_) in response to benzaldehyde. **(B)** The Δspikes/s numbers of Ba6 ORNs of WT, ds-GFP, ds-5-HT_2_, and ds-GABA_b_ locusts in response to guaiacol. **(C)** The Δspikes/s numbers of Ba6 ORNs of WT, ds-GFP, ds-5-HT_2_, and ds-GABA_b_ locusts in response to phenylacetonitrile. **(D–F)** The change of Δspikes/s numbers (represented by (ΔΔspikes/s) of Ba6 ORNs) in response to the three odorants between ds-5-HT_2_ or ds-GABA_b_ and WT or ds-GFP. Values are the mean ± SEM, *n* = 5 per group. *p < 0.05, one-way ANOVA with Tukey's *post-hoc* tests. ns, no significant difference.

We, next, examined the responses of Ba6 neurons in ds-*Lmig*5HT_2_, ds-*Lmig*GABA_b_, ds-GFP, and wild-type locusts to benzaldehyde (Figure 7A), guaiacol (Figure 7B), and phenylacetonitrile (Figure 7C) at concentrations (v/v) of 0.1%, 0.5%, 1%, 5%, and 10%.

In Figures 7A–C, when using 0.1% of the concentration of the three odorants to stimulate, only benzaldehyde cause a significantly higher response (number of Δspikes/s) of RNAi of 5-HT_2_ receptor and GABA_b_ genes (ds-5-HT_2_ and ds-GABAb) than those of WT and ds-GFP. Nevertheless, at 0.5%, 1%, 5%, and 10% concentrations, all responses of ds-5-HT_2_ and ds-GABA_b_ exhibited significantly higher responses to all three odorants. This indicated that 5-HT_2_ and GABA_b_ receptors do not function as inhibition when stimulation of odorant at low concentration, and there may be a threshold of odor concentration that they function.

Furthermore, we want to know whether there is a threshold odor concentration that affects the negative feedback provided by 5HT and GABA. In other words, does the strength of this negative feedback vary depending on the concentration of the stimulus odor? Therefore, we calculated the different values (represented by ΔΔspikes/s) between the ORNs' Δspikes in ds-5-HT_2_ and that in ds-GFP locusts using the same odor stimulus as a measure of the intensity of the 5HT negative feedback. We found that at low concentrations (0.1% to 1%) of benzaldehyde and guaiacol, ΔΔspikes/s kept stable, but as the concentrations increased, ΔΔspikes/s sharply increased to produce significant differences to low concentrations (Figures 7D, E). However, when phenylacetonitrile was used as the stimulus, ΔΔspikes/s increases with increasing concentration, with a significant difference in ΔΔspikes/s only between 0.1% and 10% concentrations (Figure 7F). The ΔΔspikes/s between ds-GABA_b_ and dsGFP locusts as a variety of compounds' concentration was compared, and the trend was similar to that of ds-5-HT_2_ (Supplementary Figure S5). This finding indicates that 5-HT/GABA has a clear inhibitory or negative modulatory role in neuronal responses elicited by concentrations of odors, which means that the higher the concentration of odors, the stronger the inhibition of 5-HT/GABA to neuronal responses in the antenna of locust.

## 4. Discussion

Our experiment demonstrated that 5-HT and GABA exist in the antenna because two key synthetic enzyme genes for 5-HT were localized in the antenna, and these two genes were detected by PCR. However, the two synthetic enzyme genes for 5-HT were localized in cells surrounding ORNs in the antenna, indicating that 5-HT may be synthesized in cells surrounding ORNs, but not ORNs. Furthermore, our experiment on the localization of the 5-HT receptor in the antenna showed that the receptor was expressed in accessory cells surrounding ORNs, whereas the receptor was expressed in the GABAb receptor gene in ORNs. Depression of the 5-HT receptor gene in isolated antennae caused similar response patterns of antennal ORNs in locusts. Therefore, 5-HT, GABA, and their receptors exist in the peripheral nervous system and are at least partially involved in the local modulation of olfaction in insect antennae. Among them, the results of the 5HT receptor's localization were consistent with the previous studies on mosquitoes that 5-HT-immunoreactive fibers were observed in the antenna (Siju et al., 2008; Pitts et al., 2011).

A majority of previous studies of 5-HT function were focused on the CNS (Hurley et al., 2004; Becnel et al., 2011; Brunert et al., 2016; Sizemore and Dacks, 2016) or throughout the nervous system (Anstey et al., 2009; Guo et al., 2013; Tanaka and Nishide, 2013). For instance, 5-HT has been proposed to regulate gregarious behavior in the desert locust, *Schistocerca gregaria* (Anstey et al., 2009). Another study suggested that 5-HT enhances solitariness in the phase transition of locusts (Guo et al., 2013). Reciprocally, Tanaka et al. have suggested that 5-HT has no effect on the attraction/avoidance behavior, which may be the most important factor in the process of gregarious behavior of desert locusts (Tanaka and Nishide, 2013). Nonetheless, these studies on 5HT function do not distinguish between the central nervous system and the peripheral nervous system. There need to be further studies on 5-HT function in the peripheral nervous system of insects because of a lack of studies on its receptors. At least nine types of 5-HT receptors have been identified throughout the various layers of the mammalian olfactory bulb (Pazos et al., 1985; Shen et al., 1993; Tecott et al., 1993; Grailhe et al., 1999; Yuan et al., 2003; Ganesh et al., 2010; Klein et al., 2012; Oba et al., 2013; Suwa et al., 2014), and five receptor classes are broadly distributed throughout multiple cell types in the insect antennal lobe (Witz et al., 1990; Saudou et al., 1992; Colas et al., 1995; Gasque et al., 2013; Sizemore and Dacks, 2016). In our study, only one 5-HT receptor gene, *Lmig*5-HT_2_, was reported. However, there might be more other putative 5-HT receptor genes (series No. MN531678, MN531680) in locust antenna when we searched by tBlast and PCR (Supplementary Figure S4), and their function is open to be studied.

Little is known about the expression of 5-HT receptors in the peripheral nervous system, particularly their relationship with ORNs and accessory cells in sensilla of the antenna. Our dual-color fluorescence *in situ* hybridization experiments showed that *Lmig*5-HT_2_ localized in accessory cells adjacent to ORNs, but not in ORNs. This is similar to its expression in mammals that 5-HT_2C_ receptor expressed in some juxtaglomerular cells in the olfactory bulb, not in ORNs (Petzold et al., 2009). In addition, our study showed that the GABA_b_ receptor was identified and localized in ORNs of antenna, which is similar to a few previous studies that solely reported GABA_b_ receptors in ORNs (Root et al., 2008; Pregitzer et al., 2013). Our localization experiment on 5-HT_2_ and GABA_b_ receptors revealed the spatial relationship between these two receptors in insect antennae, and this provides a link to understanding the pathway for 5-HT/GABA and their receptors' functions considering that 5-HT/GABA receptors were often demonstrated in the cascade in the modulation of the nervous process (Jacobs and Azmitia, 1992; Dacks et al., 2008; Kloppenburg and Mercer, 2008).

By measuring the responses of ORNs after suppressing the expression of corresponding genes, we demonstrated that the *Lmig*5-HT_2_ receptor plays an inhibitory role in the response of ORNs to odorants, which results in the regulation of the signal output. Previous studies in moths suggested that 5-HT_2_ in the sensillum, lymph might regulate neuronal activity by altering the transepithelial potential, thus changing the threshold of firing without influencing the spontaneous action potential activity of ORNs (Dolzer et al., 2001). Because ORNs performed in very similar patterns when locusts had knockdown of GABA_b_, we suggest that regulation by the *Lmig*5-HT_2_ receptor may indirectly mediate olfaction through the activation of the *Lmig*GABA_b_ receptor in the ORNs of antennae. These results indirectly supported the pathway for 5-HT/GABA and their receptors' function in a cascade too.

Our findings indicate that the depression of the 5-HT_2_ and GABA_b_ receptors increased sensory responses to odors, and in other words, the 5-HT_2_ and GABA_b_ attenuated sensory response. In addition, the depression of 5-HT_2_ and GABA_b_ receptors caused a dose-dependent response, indicating that serotonergic/GABAnergic modulation of odor input in the peripheral nervous system may be dose-dependent, and the modulation starts when the concentration of odorants over the thresholds. Another interesting result was that at higher odor concentrations, the inhibition by serotonin/GABA became stronger. Such dose-dependent modulation of ORNs during olfaction can dynamically control olfactory input. This process is similar to that of the olfactory bulbs of mammals or the antennal lobes of insects (Dacks et al., 2009; Petzold et al., 2009). However, it is the opposite of a study on moths that serotonin increased the amplitude of odor-evoked neuronal responses in the antenna (Dacks et al., 2008). It is interesting that our experiment on the isolated antenna (the antenna was cut from the body of locusts) for the first time showed that the depression of 5-HT and GABA receptors elicited an increase in responses of ORNs, indicating that the serotonergic/GABAergic modulation of odor input in the peripheral nervous system may be independent to some extent from the brain in insects.

The odorants pass through the pores on the sensillar wall and are then bound by OBPs that are secreted by accessory cells to form the odorant/OBP complex, which is transported onto ORs and OR co-receptors on the membranes of ORN dendrites, thus evoking action potentials in ORNs (Laughlin et al., 2008). Studies have shown that in the antennae of male *Manduca sexta* moths, 5-HT affects the transepithelial potential, generated by accessory cells in the olfactory sensillum and creates a driving force for the receptor current (Dolzer et al., 2001; Grosmaitre et al., 2001), whereas it has no direct effect on the activity of spontaneous action potential of olfactory receptor neurons (Dolzer et al., 2001). This is consistent with our results that the 5-HT receptor is expressed in the accessory cells but not on ORNs, which suggests that 5-HT acts directly on accessory cells to affect the ORNs when acting in the peripheral nervous system, rather than directly on ORNs. Response suppression about 5-HT might also be an important mechanism by which the brain uses 5-HT to gate out sensory information that might otherwise compete for attention or other cognitive resources (Saudou et al., 1992).

## Data availability statement

The data presented in the study are deposited in the NCBI repository, accession number PRJNA950534. The deposition data has been successfully published, linked below. https://www.ncbi.nlm.nih.gov/bioproject/PRJNA950534/; https://doi.org/10.6084/m9.figshare.22216951.v1.

## Author contributions

XZ proposed the idea. XZ, ML, and LZ designed the experiments. XX, ML, and BY conducted the experiments. XZ, ML, XX, and LZ wrote the manuscript. All authors contributed to the article and approved the submitted version.
