# Supplementary figures and images for: Serotonin/GABA receptors modulate odor input to olfactory receptor neuron in locusts
Source: Front Cell Neurosci. 2023 Apr 28;17:1156144. doi: 10.3389/fncel.2023.1156144 (PMC10175586; doi:10.3389/fncel.2023.1156144)

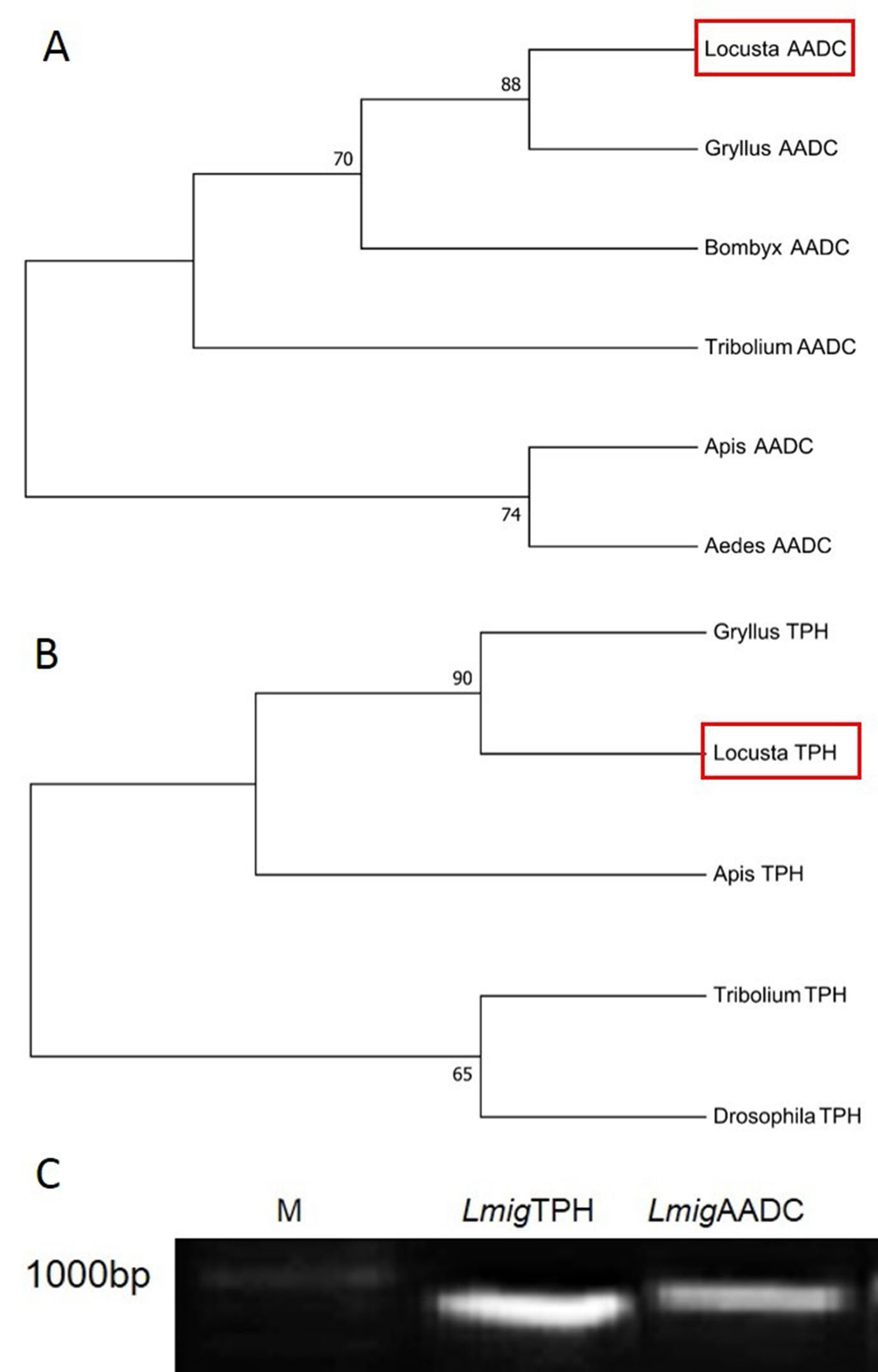

Supplement: Supplementary file 1 [file Image_1.JPEG]

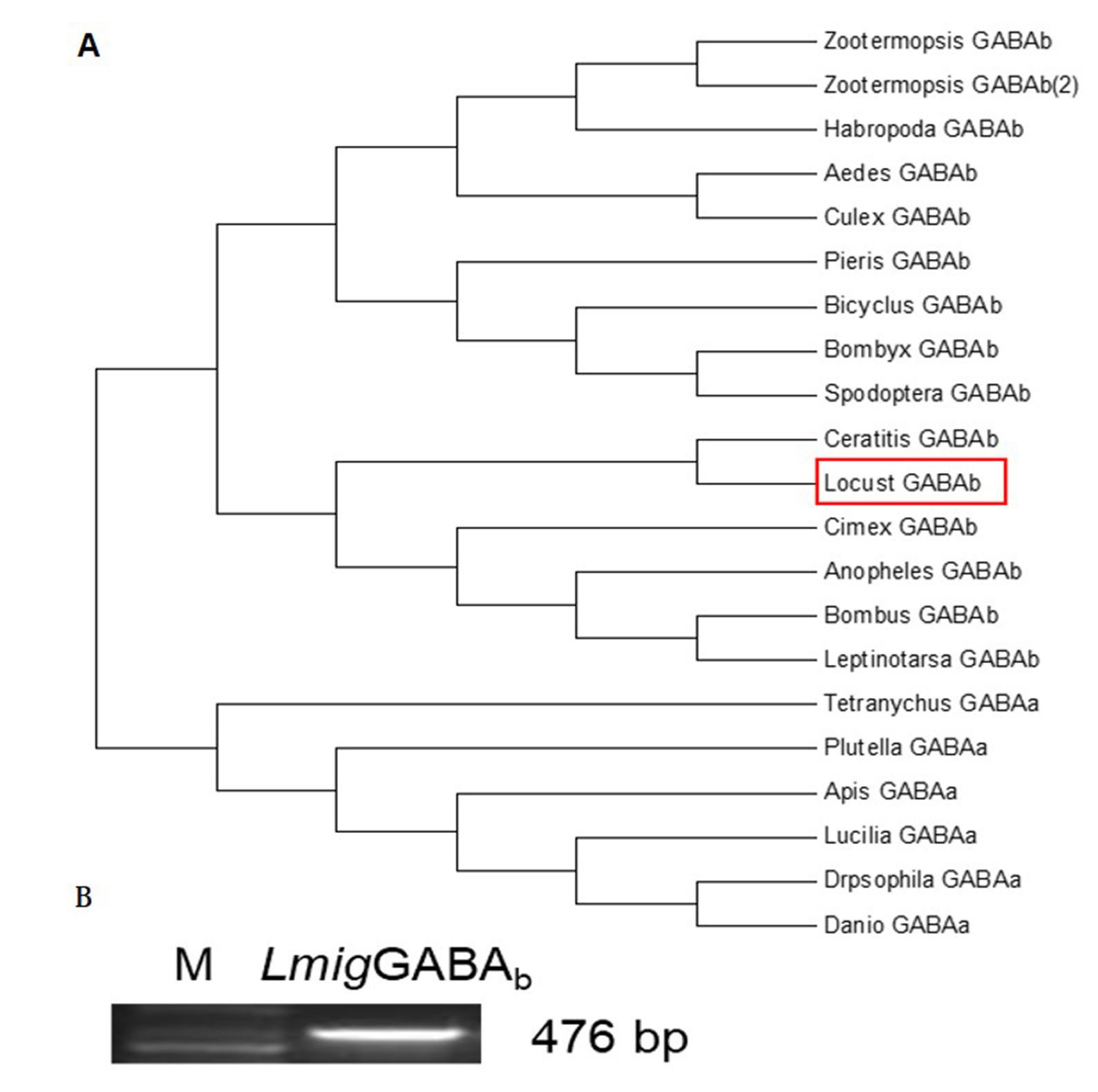

Supplement: Supplementary file 2 [file Image_2.JPEG]

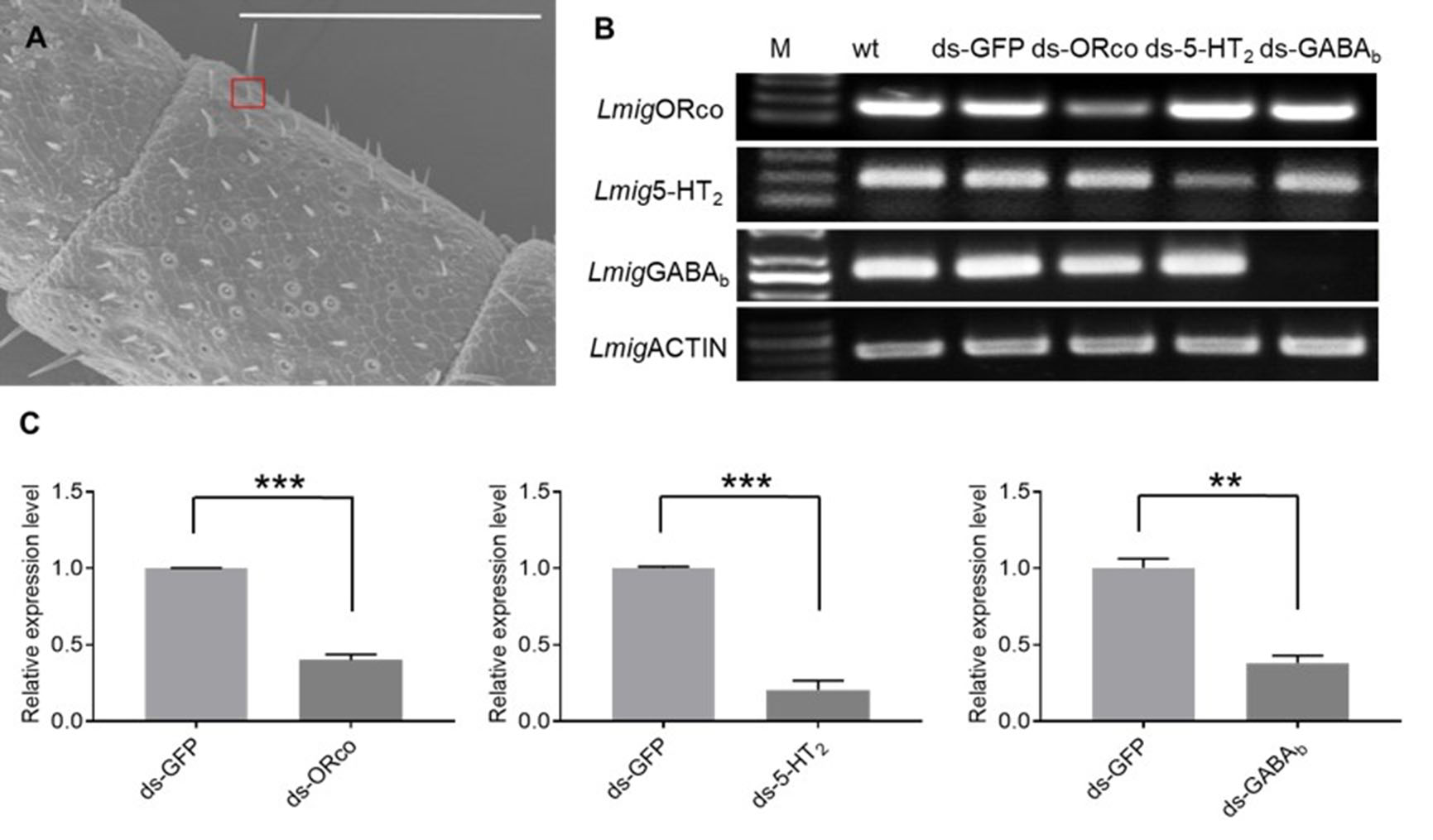

Supplement: Supplementary file 3 [file Image_3.JPEG]

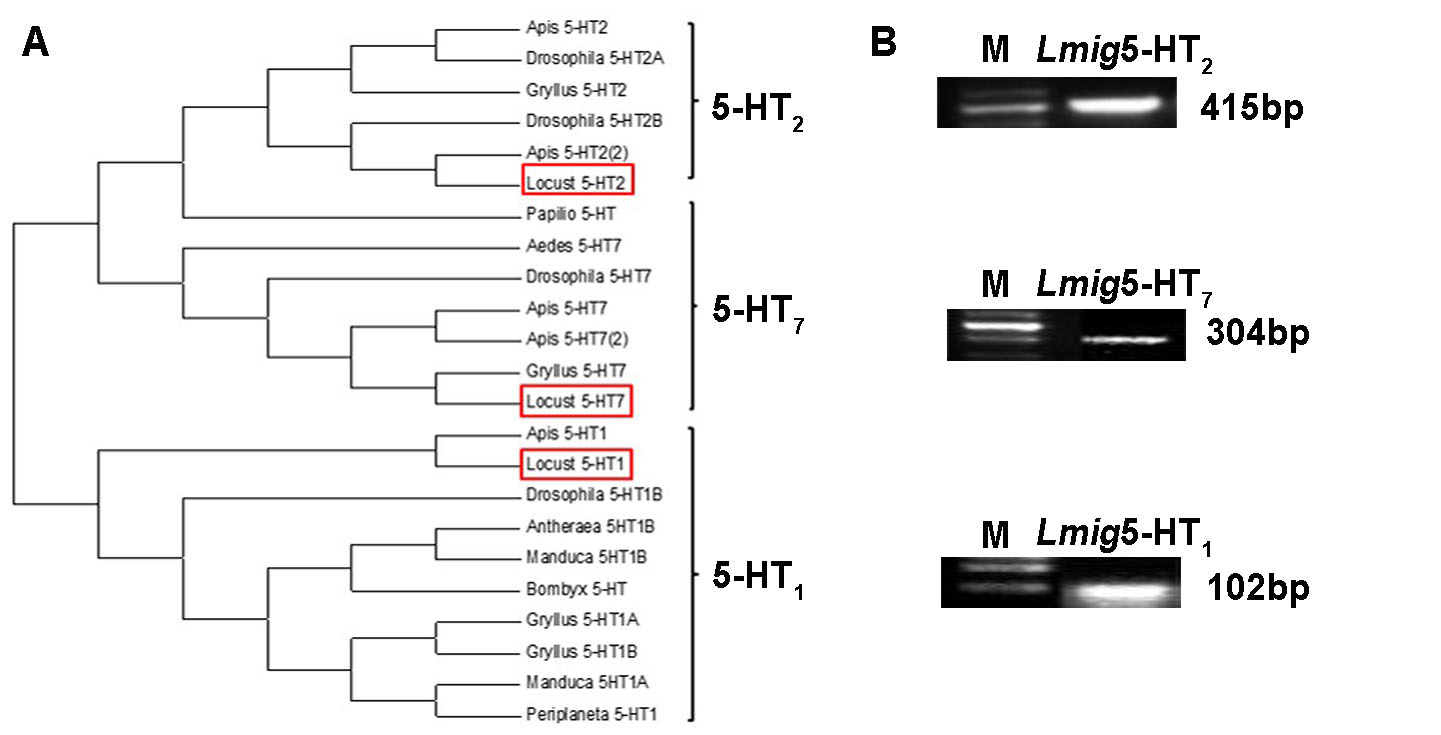

Supplement: Supplementary file 4 [file Image_4.JPEG]

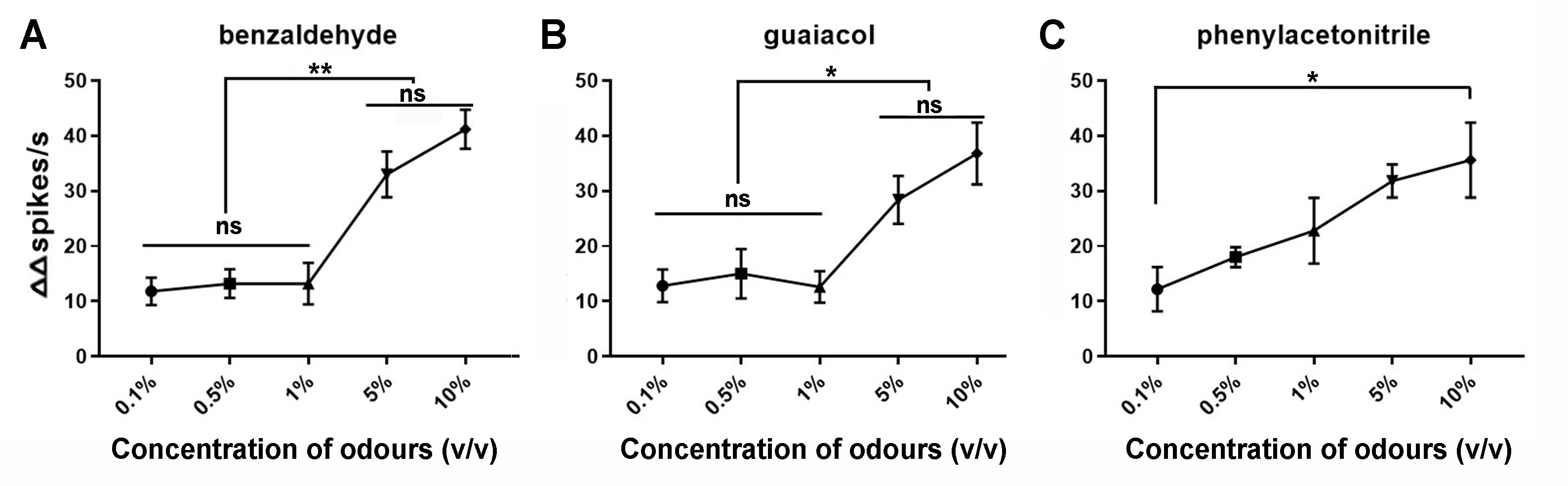

Supplement: Supplementary file 5 [file Image_5.JPEG]
