# Supplementary material for: Serotonin/GABA receptors modulate odor input to olfactory receptor neuron in locusts
Source: Front Cell Neurosci. 2023 Apr 28;17:1156144. doi: 10.3389/fncel.2023.1156144 (PMC10175586; doi:10.3389/fncel.2023.1156144)
Supplement: Supplementary file 6 [file Table_1.docx]

**TABLE S1. 5-HT receptors of other insect species were used for the phylogenetic tree.**

| **Gene bank #** | **Insect** | **Gene** |
| --- | --- | --- |
| AF296125.2 | *Aedes aegypti* | 5-HT7 |
| EU402613.1 | *Antheraea pernyi* | 5-HT1B |
| AM076717.1 | *Apis mellifera* | 5-HT7 |
| FR727108.1 | *Apis mellifera* | 5-HT2B |
| FR727107.1 | *Apis mellifera* | 5-HT2A |
| FN645449.1 | *Apis mellifera* | 5-HT1 |
| NM_001077821.1 | *Apis mellifera* | 5-HT7 |
| NM_001044037.1 | *Bombyx mori* | 5-HT |
| NM_079860.3 | *Drosophila melanogaster* | 5-HT7 |
| NM_141549.4 | *Drosophila melanogaster* | 5-HT2B |
| NM_169011.3 | *Drosophila melanogaster* | 5-HT2A |
| NM_079065.6 | *Drosophila melanogaster* | 5-HT1B |
| AB618101.1 | *Gryllus bimaculatus* | 5-HT7 |
| AB618100.1 | *Gryllus bimaculatus* | 5-HT2α |
| AB618099.1 | *Gryllus bimaculatus* | 5-HT1B |
| AB618098.1 | *Gryllus bimaculatus* | 5-HT1A |
| DQ840516.1 | *Manduca sexta* | 5-HT1B |
| DQ840515.1 | *Manduca sexta* | 5-HT1A |
| AB182632.1 | *Papilio xuthus* | 5-HT |
| FN298392.1 | *Periplaneta americana* | 5-HT1 |

**TABLE S2. GABA receptors of other insect species were used for the phylogenetic tree.**

| **Gene bank #** | **Insect** | **Gene** |
| --- | --- | --- |
| XP_021704022.1 | *Aedes aegypti* | GABA_b_ |
| XP_049537915.1 | *Anopheles darlingi* | GABA_b_ |
| XP_006565169.1 | *Apis mellifera* | GABA_a_ |
| XP_052739444.1 | *Bicyclus anynana* | GABA_b_ |
| XP_012176171.1 | *Bombus terrestris* | GABA_b_ |
| XP_012548554.3 | *Bombyx mori* | GABA_b_ |
| XP_012160499.1 | *Ceratitis capitata* | GABA_b_ |
| XP_014251512.1 | *Cimex lectularius* | GABA_b_ |
| EDS35744.1 | *Culex quinquefasciatus* | GABA_b_ |
| NP_001261616.1 | *Drosophila melanogaster* | GABA_a_ |
| KOC69015.1 | *Habropoda laboriosa* | GABA_b_ |
| XP_023016883.1 | *Leptinotarsa decemlineata* | GABA_b_ |
| AAB81966.1 | *Lucilia cuprina* | GABA_a_ |
| XP_022123923.2 | *Pieris rapae* | GABA_b_ |
| NP_001292464.1 | *Plutella xylostella* | GABA_a_ |
| BAJ41377.1 | *Tetranychus urticae* | GABA_a_ |
| XP_015840082.1 | *Tribolium castaneum* | GABA_a_ |
| XP_022814536.1 | *Spodoptera litura* | GABA_b_ |
| KDR18733.1 | *Zootermopsis nevadensis* | GABA_b_ |
| XP_021921382.1 | *Zootermopsis nevadensis* | GABA_b_ |

**TABLE S3. LPH and AADC of other insect species were used for the phylogenetic tree.**

| **Gene bank #** | **Insect** | **Gene** |
| --- | --- | --- |
| XM_623297.5 | *Apis mellifera* | TPH |
| XM_961932.4 | *Tribolium castaneum* | TPH |
| NM_079239.4 | *Drosophila melanogaster* | TPH |
| AB618096.1 | *Gryllus bimaculatus* | TPH |
| XM_001648213.1 | *Aedes aegypti* | AADC |
| NM_001043709.1 | *Bombyx mori* | AADC |
| XM_394115.6 | *Apis mellifera* | AADC |
| NM_001102586.1 | *Tribolium castaneum* | AADC |
| AB618097.1 | *Gryllus bimaculatus* | AADC |

**TABLE S4. Primers for PCR, RNAi, and *in situ* hybridisation experiments.**

**
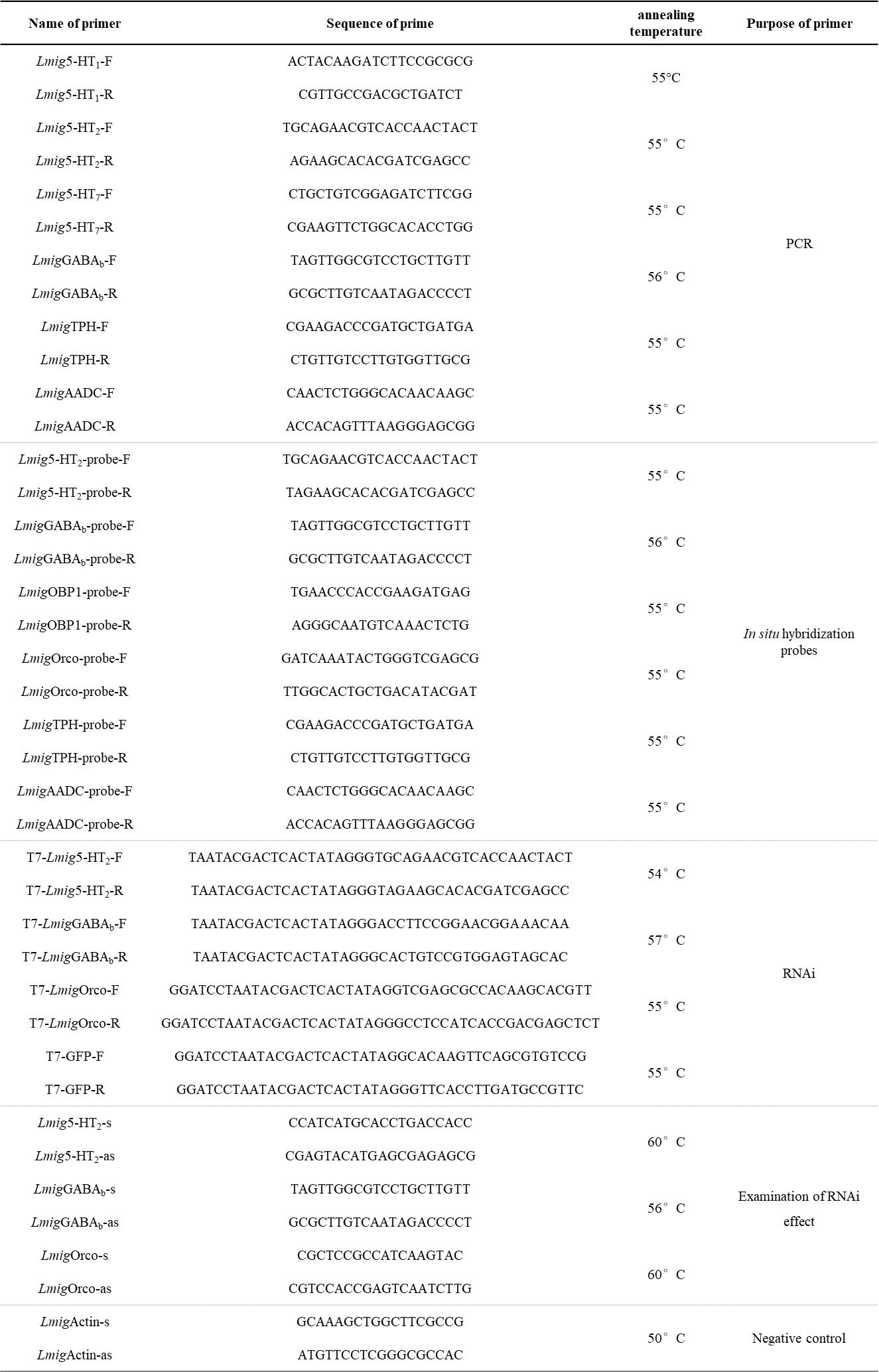
**

**TABLE S5. Odorants that were used in the electrophysiological experiments.**

| Odorant | Molecular Formula | CAS | Lot Number |
| --- | --- | --- | --- |
| Trans-2-Hexnyl acetate | C_8_H_14_O_2_ | 2497-18-9 | 103004-10G, SIGMA-ALDRICH |
| Benzaldehyde | C_7_H_6_O | 100-52-7 | B1334-250ML, SIGMA-ALDRICH |
| Guaiacol | C_7_H_8_O_2_ | 90-05-1 | 120192500-250G, ACROS ORGANICS |
| Phenylacetonitrile | C_8_H_7_N | 140-29-4 | 13300-250ML, Fluka |
